# Antiprotozoal Activity of *Buxus sempervirens* and Activity-Guided Isolation of *O*-tigloylcyclovirobuxeine-B as the Main Constituent Active against *Plasmodium falciparum* ‡

**DOI:** 10.3390/molecules19056184

**Published:** 2014-05-15

**Authors:** Julia B. Althaus, Gerold Jerz, Peter Winterhalter, Marcel Kaiser, Reto Brun, Thomas J. Schmidt

**Affiliations:** 1 Institut für Pharmazeutische Biologie und Phytochemie (IPBP), University of Münster, Pharma Campus, Corrensstraße 48, D-48149 Münster, Germany; E-Mail: julia.althaus@uni-muenster.de; 2 Institut für Lebensmittelchemie, Technische Universität Braunschweig, Schleinitzstraße 20, D-38106 Braunschweig, Germany; E-Mails: g.jerz@tu-bs.de (G.J.); p.winterhalter@tu-bs.de (P.W.); 3 Tropical and Public Health Institute (Swiss TPH), Socinstraße 57, CH-4002 Basel, Switzerland; E-Mails: Marcel.Kaiser@unibas.ch (M.K.); Reto.Brun@unibas.ch (R.B.); 4 University of Basel, Petersplatz 1, CH-4003 Basel, Switzerland

**Keywords:** *Buxus sempervirens*, antiprotozoal activity, cycloartane alkaloids, *Plasmodium falciparum*, *Trypanosoma brucei rhodesiense*, *Trypanosoma cruzi*, *Leishmania donovani*, spiral-coil countercurrent chromatography

## Abstract

*Buxus sempervirens* L. (European Box, Buxaceae) has been used in ethnomedicine to treat malaria. In the course of our screening of plant extracts for antiprotozoal activity, a CH_2_Cl_2_ extract from leaves of *B. sempervirens* showed selective *in vitro* activity against *Plasmodium falciparum* (IC_50_ = 2.79 *vs.* 20.2 µg/mL for cytotoxicity against L6 rat cells). Separation of the extract by acid/base extraction into a basic and a neutral non-polar fraction led to a much more active and even more selective fraction with alkaloids while the fraction of non-polar neutral constituents was markedly less active than the crude extract. Thus, the activity of the crude extract could clearly be attributed to alkaloid constituents. Identification of the main triterpene-alkaloids and characterization of the complex pattern of this alkaloid fraction was performed by UHPLC/+ESI-QTOF-MS analyses. ESI-MS/MS target-guided larger scale preparative separation of the alkaloid fraction was performed by ‘spiral coil-countercurrent chromatography’. From the most active subfraction, the cycloartane alkaloid *O*-tigloylcyclovirobuxeine-B was isolated and evaluated for antiplasmodial activity which yielded an IC_50_ of 0.455 µg/mL (cytotoxicity against L6 rat cells: IC_50_ = 9.38 µg/mL). *O*-tigloylcyclovirobuxeine-B is thus most significantly responsible for the high potency of the crude extract.

## 1. Introduction

*Buxus sempervirens* L. (European Box; Buxaceae) is known in the Mediterranean area as a plant with antimalarial activity [1,2,3]. Ethnomedicinal studies show that populations from geographically isolated areas have independently acquired knowledge about the therapeutical use of box plants against malaria. The form of application reported in two independent studies is a decoction of the leaves [1,2]. Other *Buxus* species have also been used for the same purpose on other continents [4].

The genus *Buxus* and *B. sempervirens* in particular are known to be a rich source of different types of triterpene alkaloids. In the literature, this type of natural products are also termed steroidal alkaloids [4,5]. In the course of our ongoing search for secondary plant metabolites with anti‑protozoan activity [6,7,8,9,10], a dichloromethane extract of *B. sempervirens* leaves showed significant and selective *in vitro* activity against the NF54 strain of *Plasmodium falciparum* (*Pf*), the etiologic agent of tropical malaria [11]. The primary aim of this work was therefore to identify the compound(s) responsible for this antiplasmodial effect using a bioactivity-guided fractionation protocol. The extract and fractions were also tested for activity against *Trypanosoma brucei rhodesiense* (*Tbr*, East African sleeping sickness), *T. cruzi* (*Tc*, Chagas disease) and *Leishmania donovani* (*Ldo*, visceral leishmaniasis, Kala-Azar).

## 2. Results and Discussion

### 2.1. Antiprotozoan Activity of B. sempervirens Leaf Extract and the Alkaloid Fraction

The dichloromethane extract of *B. sempervirens* leaves was investigated for *in vitro* activity against *Tbr*, *Tc*, *Ldo* and *Pf* and found to be selectively active against the NF54 strain of *Pf* with an IC_50_ value of 2.79 µg/mL and a selectivity index (SI, ratio of IC_50_ value for cytotoxicity against L6 rat skeletal myoblasts over IC_50_ for antiparasitic activity) of approximately 7 (see Table 1). Since *Buxus* species are known to contain a rich variety of triterpene alkaloids, the extract was partitioned into a fraction containing basic compounds and a non-polar neutral part. Extraction of a solution with diluted H_2_SO_4_ followed by neutralization and extraction with CH_2_Cl_2_ yielded about 25% of a residue consisting of basic (alkaloid) compounds. Re-evaluation in the bioassay revealed that the antiplasmodial activity had been concentrated in the alkaloid fraction (ALK). It displayed an IC_50_ value of 0.36 µg/mL against *Pf* while the non-polar neutral fraction (APO) was much less active (IC_50_= 7.8 µg/mL). Moreover, the selectivity of the alkaloid fraction was much higher (SI = 20.4) than that of the neutral fraction (SI = 4.4) or that of the crude extract.

**Table 1:** IC_50_ values of *B. sempervirens* leaf extract, its fractions and subfractions as well as isolated *O*-tigloylcyclovirobuxeine B (**1**). All data represent the mean of at least two independent determinations and are expressed in µg/mL, those for **1** and the positive controls also in µM (in brackets).

Tested sample	*Pf*	*Tbr*	*Tc*	*Ldo*	Cytotox. L6
chloroquine	0.002 ± 0.000 (0.006 ± 0.000)				
melarsoprol		0.004 ± 0.001 (0.010 ± 0.003)			
benznidazole			0.607 ± 0.019 (2.332 ± 0.073)		
miltefosine				0.075 ± 0.005 (0.184 ± 0.012)	
podophyllotoxin					0.007 ± 0.002 (0.017 ± 0.005)
extract	2.79 ± 0.39	>10	>10	>10	20.2 ± 1.3
APO	7.76 ± 1.21	n.d.	n.d.	n.d.	33.9 ± 3.7
ALK	0.361 ± 0.019	n.d.	n.d.	n.d.	7.31 ± 0.42
spCCC subfractions elution mode					
62	2.94 ± 0.09	1.49 ± 0.20	n.d.	n.d.	10.75 ± 0.75
68	3.32 ± 0.08	1.10 ± 0.51	n.d.	n.d.	6.98 ± 1.02
82	2.85 ± 0.06	1.61 ± 0.33	n.d.	n.d.	7.47 ± 1.66
98	2.40 ± 0.09	0.246 ± 0.01	n.d.	n.d.	2.65 ± 0.02
116	1.58 ± 0.00	0.181 ± 0.02	n.d.	n.d.	2.53 ± 0.13
130	1.32 ± 0.00	0.215 ± .01	n.d.	n.d.	3.68 ± 0.17
142	1.37 ± 0.20	0.222 ± 0.01	n.d.	n.d.	3.73 ± 0.14
146	1.53 ± 0.39	0.411 ± 0.19	n.d.	n.d.	4.24 ± 0.33
spCCC subfractions extrusion mode					
E9	1.03 ± 0.02	0.126 ± 0.06	n.d.	n.d.	4.80 ± 0.55
E53	0.861 ± 0.03	0.945 ± 0.25	n.d.	n.d.	14.00 ± 2.40
E73	0.787 ± 0.24	0.381 ± 0.10	n.d.	n.d.	12.90 ± 1.10
E97	7.88 ± 0.37	2.79 ± 0.73	n.d.	n.d.	37.73 ± 10.77
E105	8.42 ± 0.19	2.50 ± 0.18	n.d.	n.d.	55.50 ± 1.90
compound **1** (µM)	0.455 ± 0.169 (0.917 ± 0.341)	1.83 ± 0.05 (3.68 ± 0.101)	13.49 ± 7.31 (27.20 ± 14.73)	85.35 ± 7.75 (172.08 ± 15.63)	9.38 ± 4.12 (18.91 ± 8.31)

### 2.2. Bioactivity-Guided Separation of the Alkaloid Fraction

In order to identify the compound(s) responsible for the high activity and selectivity, the alkaloid fraction was submitted to larger lab-scale preparative “spiral-coil countercurrent chromatography” (spCCC) and off-line ESI-MSMS-target-guided injection analysis. For this purpose, 7.4 g of the alkaloid fraction were submitted to spCCC. An advantage of this all-liquid chromatography method is that chemisorption effects with a potential loss of relevant bioactive compounds can be completely avoided. From this separation, 126 subfractions were obtained of which every second was analyzed by direct injection by sequence of recovery to the ion trap +ESI-MSMS. Every injection peak displayed the full mass-spectrometric metabolite profile present in a specific collected fraction and enabled the immediated molecular weight characterization of eluting compounds. Based on these mass spectrometric results, subfractions containing all representative alkaloids were selected and tested for their *in vitro* antiprotozoal activity in a growth inhibition assay (Table A1, appendix). The IC_50_ values of these fractions against *Pf* and *Tbr* are reported along with their cytotoxicity data in Table 1.

**Figure 1:**
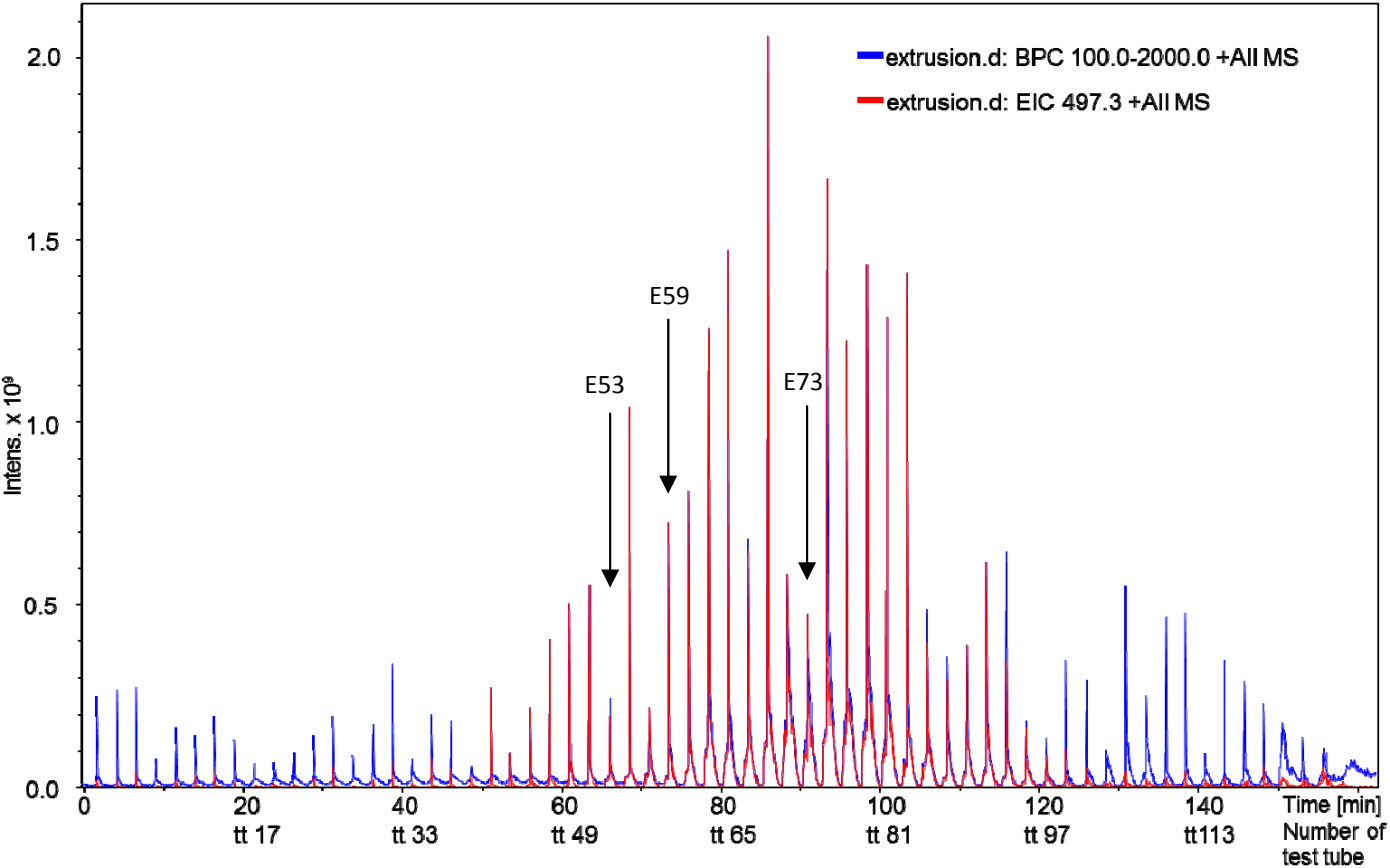
Mass spectrometric (direct injection ion trap +ESI MSMS) monitoring of spCCC fractions obtained in extrusion mode. Every second test tube (axis lable TT) was analyzed. Red: extracted target ion trace of *m/z* = 497, Blue: base peak chromatogram of *m/z* 100–2000. Arrows: Subfractions E53 and E73 as well as E59 shown in Figure 2. Compound **1** was isolated from the pooled subfractions E54–E69.

**Figure 2:**
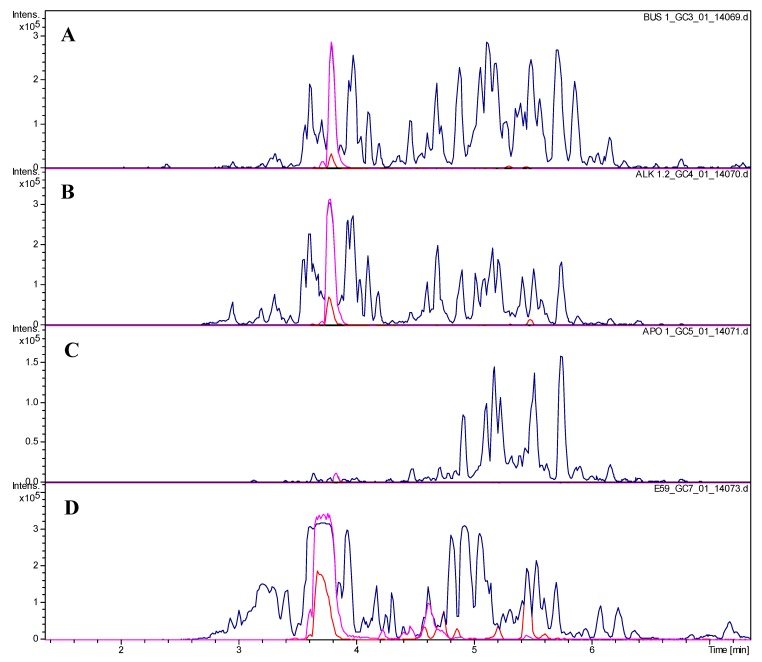
UHPLC/+ESI-QTOF-MS chromatograms of (**A**) the crude extract, (**B**) the alkaloid fraction ALK, (**C**) the non-polar neutral fraction APO and (**D**) of subfraction E 59. Blue: Base peak chromatogram of *m/z* 100–1000, Red: Extracted ion chromatogram of *m/z* 497 [M+H]^+^; Magenta: extracted ion chromatogram of *m/z* 249 [M+2H]^2+^.

The most active of the tested fractions against *Pf*, E53 and E73 of the extrusion phase, with IC_50_ values below 1 µg/mL were found by +ESI-QTOF-MSMS to contain one major alkaloid with a molecular mass of 496 Da which is a main constituent of the total extract (see Figure 1 and Figure 2). From the extruded spCCC fraction E73, this alkaloid (**1**) was purified by preparative HPLC.

### 2.3. Identification and Full Spectroscopic Characterization of O-tigloylcyclovirobuxeine-B *(**1**)*

**Figure 3:**
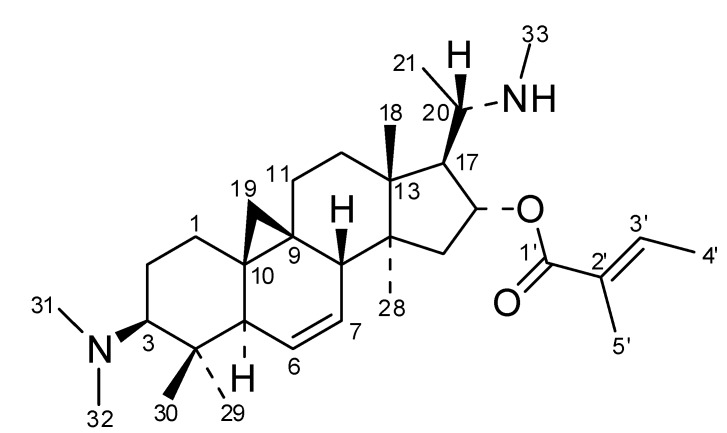
Structure of *O*-tigloylcyclovirobuxeine-B (**1**).

**Figure 4:**
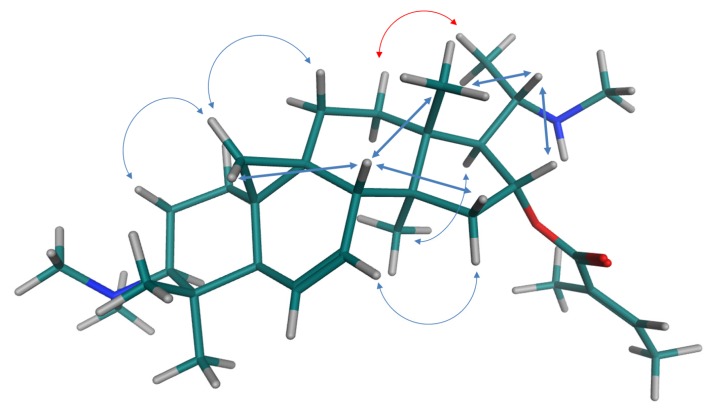
Main NOEs observed in a 2D-NOESY spectrum of *O*-tigloylcyclovirobuxeine-B (**1**). The molecular model represents the lowest energy conformer found in a conformational search.

The +ESI-QTOF mass spectrum of compound **1** displayed quasimolecular ions at *m/z* 249.2096 representing a doubly charged proton adduct [M+2H]^2+^ and at *m/z* 497.4105 [M+H]^+^ which showed the presence of an alkaloid containing two basic nitrogen atoms. The molecular formula of **1** could thereby be established as C_32_H_52_N_2_O_2_. Its NMR spectra (^1^H, ^13^C, ^1^H/^1^H-COSY, ^1^H/^13^C-HSQC and HMBC as well as ^1^H/^1^H-NOESY; full data see Table 2, all spectra are shown in the appendix) showed the presence of a hexanorcycloartane derivative with the characteristic signals of the cyclopropane-methylene group CH_2_-19 at δ_H_-0.18 and 0.72 ppm (both d, 4 Hz) and δ_C_ 18.2 ppm. Further characteristic signals were assigned a dimethylamino-(δ_H_ 2.28 s, 6H) and a further methylamino-(δ_H_ 2.34, s, 3H) substituent as well as a tiglic acid ester moiety (δ_H_ 6.83, qq; 1.81 dq and 1.76, dq). Full analysis of its HMBC spectrum established all atom connectivities and the NOESY spectrum allowed assignment of the relative stereochemistry, altogether leading to the unambiguous identification of compound **1** as *O*-tigloylcyclovirobuxeine-B (Figure 3). The configuration at C-20 was determined as depicted and in accordance with the previous assignment [12,13] (*i.e.*, *S) by an NOE-effect between CH_3_-21 and H-12β (red arrow in Figure 4) which would not be possible in the *R configuration, as deduced from molecular models obtained by a conformational search.

**Table 2:** NMR data of *O*-tigloylcyclovirobuxeine-B (**1**) in CDCl_3_. All assignments were confirmed by ^1^H/^13^C-HSQC and -HMBC correlations.

Position	^1^H-NMR			^13^C NMR
	δ (ppm)	mult.	J (Hz)	δ (ppm)
1	1.53	* (2H)		31.19
2	1.75 1.53	* *		20.01
3	2.029	dd	3.7; 11.4	71.50
4				41.60
5	1.83	*		48.88
6	5.604	ddd [dt]	1.3; 1.3; 10.6	128.01
7	5.366	ddd	3.1; 6.1; 10.6	128.16
8	2.568	dd	1.9; 6.1	43.16
9				20.74
10				28.78
11	1.85 1.44	* *		25.13
12	1.74 1.49	* *		32.31
13				46.11
14				49.53
15	2.140 1.235	dd d(d)	8.2; 14.3 14.3; (<1)	42.80
16	5.095	ddd	1.1; 6.1; 8.5	80.47
17	2.115	dd	6.1; 9.6	57.11
18	0.995	s (3H)		15.99
19	0.716 −0.182	d d	4.1 4.1	18.22
20	2.628	dq	9.7; 6.1	57.40
21	1.077	d (3H)	6.1	18.66
28	0.941	s (3H)		17.59
29	1.028	s (3H)		26.17
30	0.782	s (3H)		16.61
31/32	2.283	s (6H)		44.30
33	2.340	s (3H)		33.48
1’				167.99
2’				137.63
3’	6.837	qq	1.5; 7.1	128.93
4’	1.762	dq (3H)	7.1; 1.2	14.54
5’	1.808	dq [quin] (3H)	1.2	12.17

* Multiplicity not determined due to signal overlap; δ-values extracted from HSQC spectrum.

This alkaloid was first isolated from *B. sempervirens* and described by Kupchan and coworkers in 1967 [12]. Since the spectroscopic data available in the literature were sparse, the full assignment of its NMR data is reported in Table 2 and Figure 4 shows the main NOESY correlations leading to its conclusive identification.

### 2.4. Antiplasmodial Activity of O-tigloylcyclovirobuxeine-B *(**1**)*

The *in vitro* antiplasmodial activity of *O*-tigloylcyclovirobuxeine-B (**1**) was evaluated after its full purification. The IC_50_ value against *Pf* was 0.455 µg/mL (0.92 µM) and it also displayed considerable selectivity (IC_50_ for cytotoxicity: 9.38 µg/mL = 18.9 µM, SI = 21; data for *Tbr*, *Tcr* and *Ldo* see Table 1). Even though the potency of the pure alkaloid was thus found slightly lower than that determined with the total alkaloid fraction, it can be assumed that compound **1** is mainly responsible for the antiplasmodial activity of *B. sempervirens* leaf extract.

### 2.5. Occurrence of O-tigloylcyclovirobuxeine-B *(**1**)* in a Decoction as Used in Ethnomedicine

**Figure 5:**
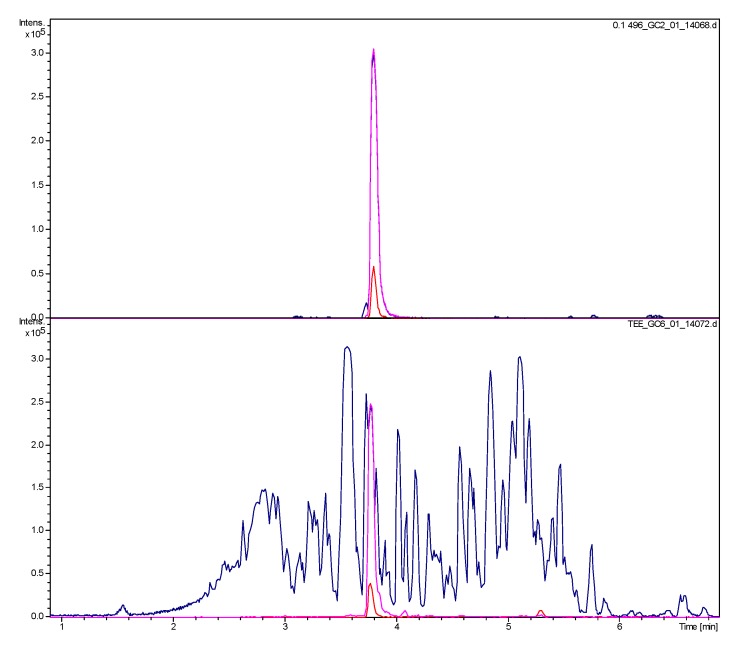
UHPLC/+ESI-QTOF-MSMS analysis of *O*-tigloylcyclovirobuxeine-B (**1**) (top) and of an aqueous decoction as used in ethnomedicine (bottom). Blue: Base peak chromatogram (*m/z* 100–1000); Red: EIC *m/z* 497 [M+H]^+^; Magenta: EIC *m/z* 249 [M+2H]^2+^.

Since *B. sempervirens* leaves have reportedly been used in the form of an aqueous decoction to treat Malaria [1,2], we investigated such a preparation for the presence of *O*-tigloylcyclovirobuxeine-B (**1**) by UHPLC/+ESI-QTOF-MSMS. The result is shown in Figure 5. The compound was clearly detectable at a significant concentration in the tea preparation. The chromatogram of pure **1** was obtained with a solution of 0.1 mg/mL while that of the tea contained 5 mg of the residue of the tea preparation corresponding to the content of a small cup (158 mL) of tea. The peak of **1** in the sample of the decoction has about 2/3 of the area of that in the chromatogram of the pure compound (estimated by integration of the EIC at *m/z* 497) which would correspond to about 70 µg of **1**. Thus, the amount applied with 1 L of tea would be in the range of 440 µg of **1**. Although the mentioned amount is a rough estimate and not the result of a validated quantitative analysis, and although we do not presently have any data on the bioavailability and pharmacokinetics of this alkaloid, this amount is certainly not insignificant, taken that repeated application of the tea was probably necessary for a cure.

## 3. Experimental Section

### 3.1. Plant Material

The aerial parts of *B. sempervirens* L. were collected in May 2012 in Havixbeck, Germany. The plant population has a half-shaded habitat. The plant was identified by T. J. Schmidt and a voucher specimen was deposited at the herbarium of IPBP, Münster (# TS_Bux_01). The plant material consisted of leaves and young stems with little lignification. The material was air dried at room temperature and ground with an IKA MF basic mill to the riddle mesh size of 1 mm.

### 3.2. Extraction Methods

#### 3.2.1. Tea Preparation based on Ethnopharmacological Reports

For the decoction, air dried leaves and young stems (3.4 g) were covered with boiling water (500 mL). After 20 min the decoction was separated though a filter paper from the plant material. The aqueous solution was extracted with dichloromethane (500 mL) and the organic phase evaporated to dryness. This extraction method yielded 15.79 mg of extract. Five mg of the extract were dissolved in 1.0 mL acetonitrile with 0.1% trifluoroacetic acid and analyzed by UHPLC/+ESI-QTOF-MS.

#### 3.2.2. Soxhlet Extraction and Acid/Base Extraction

The powdered plant material (295.5 g) was extracted exhaustively in a Soxhlet-apparatus for about 15 h with dichloromethane (2250 mL) until the supernatant was colorless. After evaporation to dryness, the extract yield was 34.28 g (11.6%). In a following acid/base extraction the content of the active compounds (fraction ALK) were enriched. The extract was dissolved in portions of 1 g in 40 mL of dichloromethane. Each portion was extracted seven times in a separatory funnel with 15 mL of diluted sulfuric acid R (1 M, European Pharmacopoeia Reagent). The combined lipophilic phases after evaporation yielded the apolar fraction (APO 22.28 g). The combined aqueous phases were neutralized with solid sodium hydroxide (≈pH 7) and subsequently extracted five times with dichloromethane (200 mL). The combined organic phases after evaporation yielded 8.72 g of the alkaloid fraction (ALK).

### 3.3. Isolation and Analytical Characterization of O-tigloylcyclovirobuxeine-B *(**1**)*

#### 3.3.1. Subfractionation by Spiral-Coil Counter Current Chromatography (Elution and Extrusion Mode)

Larger lab-scale preparative separation of the alkaloid fraction (7.4 g) was performed by spiral coil‑countercurrent chromatography (spCCC) in the ‘head-to-tail’ elution mode using the biphasic solvent system n-hexane: ethanol: water [6:5:1(v/v/v)] and pumping the aqueous lower layer as mobile phase. The required alkaloid distribution in this biphasic solvent system was evaluated before by LC‑ESI-MS analysis (data not presented).

The spCCC-prototype system (Pharma Tech Resarch, Baltimore, MD, USA) was operated at a velocity of 270 rpm, the injection concentration was 7.4 g/150 mL, flow rate of mobile phase for the elution mode was set to 15.0 mL/min. After terminating the elution process, the two solvent phases on the spCCC system containing apolar alkaloids were also recovered in tube fractions by flushing them out with nitrogen gas (extrusion mode). Both, the eluted and extruded fractions were collected in test tubes at intervals of 3 min (=45 mL). The used CCC separation column is a convoluted shaped teflon tube (ID: 8.1 mm, length 850 cm). Four serially arranged tubes are held in one separation disk, and ten disks are sequentially connected in the whole coil column system with a total volume of 5.7 L. The ESI-MSMS target profiling of spCCC fractions was performed on an HCT Ultra ETD II (ion-trap MS, Bruker Daltonics, Bremen, Germany). For these analyses, aliquots of 500 µL of recovered fractions (elution- and extrusion mode) were diluted 1:1 with acetonitrile and filled to HPLC vials. Then, the fractions were injected in the original chromatographic recovery sequence using 0.4 µL/sample by an autosampler in time intervals of 2 min, and every second test tube was analyzed.

#### 3.3.2. Purification by Preparative High Performance Liquid Chromatography (HPLC/UV-DAD)

The preparative HPLC isolation was performed on a Jasco (Groß-Umstadt, Germany) prep. HPLC system (pump: PU-2087 plus; diode array detector MD 2018 plus; column thermostat CO 2060 plus; autosampler AS 2055 plus; LC Net II ADC Chromatography Data Solutions; sample injection loop: 2000 µL) on a Reprosil 100 C-18 column (5 µm, 250 × 20mm) column with a binary gradient (A: water with 0.1% trifluoroacetic acid; B: acetonitrile with 0.1% trifluoroacetic acid) at 15mL/min with: 0 to 5 min: linear from 5% B to 20% B; 5 to 14 min: linear 20% B to 32%B; 14 min to 22 min: linear from 32% B to 35% B; 22 min to 30 min: linear 35% B to 100% B; 30 min to 35 min: isocratic 100% B. 32 mg of subfractions E54-E69 were dissolved in a concentration of 10 mg/mL acetonitrile and injected in portions of 100, 200, 300 (2×), 400 (2×), 500 and 1000 µL; Compound **1** under these conditions eluted with a retention time of 20.7 min (injection volume 500 µL). 32 mg of the subfraction led to 6.2 mg of pure *O*-tigloylcyclovirobuxeine-B (**1**).

#### 3.3.3. Analytical Profiling of the Isolation Process by UHPLC/+ ESI-QTOF-MSMS

Chromatographic separations were performed on an Ultimate 3000 RS Liquid Chromatography System (Dionex Germany, Idstein, Germany) on a Dionex Acclaim RSLC 120, C18 column (2.1 × 100 mm, 2.2 µm) with a binary gradient (A: water with 0.1% formic acid; B: acetonitrile with 0.1% formic acid) at 0.8 mL/min: 0 to 9.5 min: linear from 5% B to 100% B; 9.5 to 12.5 min: isocratic 100% B; 12.5 to 12.6 min: linear from 100% B to 5% B; 12.6 to 15 min: isocratic 5% B. The injection volume was 2 µL. Eluted compounds were detected using a Dionex Ultimate DAD-3000 RS over a wavelength range of 200–400 nm and a Bruker Daltonics micrOTOF-QII time-of-flight mass spectrometer equipped with an Apollo electrospray ionization source in positive mode at 5 Hz over a mass range of *m/z* 50–1000 using the following instrument settings: nebulizer gas nitrogen, 5 bar; dry gas nitrogen, 9 L/min, 220 °C; capillary voltage 4500 V; end plate offset −500 V; transfer time 70 µs; collision gas nitrogen; collision energy and collision RF settings were combined to each single spectrum of 1000 summations as follows: 250 summations with 20% base collision energy and 130 Vpp + 250 summations with 100% base collision energy and 500 Vpp + 250 summations with 20% base collision energy and 130 Vpp + 250 summations with 100% base collision energy and 500 Vpp. Base collision energy was 50 eV for precursor ions with a *m/z* less than 500 and then linearly interpolated against *m/z* up to a maximum of 70 eV for precursor ions with a *m/z* of up to 1000. Internal dataset calibration (HPC mode) was performed for each analysis using the mass spectrum of a 10 mM solution of sodium formiate in 50% isopropanol that was infused during LC reequilibration using a divert valve equipped with a 20 µL sample loop. Sample concentrations: The crude extract was injected at a concentration of 5 mg/mL, fractions at 0.5 mg/mL; The pure alkaloid **1** for Figure 3 was analyzed at 0.1 mg/mL. The retention time of compound **1** under these conditions was 3.8 min.

#### 3.3.4. NMR Spectroscopy

Nuclear magnetic resonance spectra were recorded on an AS 400 Mercury plus spectrometer (Varian, Palo Alto, CA, USA) at 400 (^1^H) and 100 MHz (^13^C) CDCl_3_ at room temperature. They were referenced to the solvent signals (δ_H_ 7.260 ppm, δ_C_ 77.000 ppm).

#### 3.3.5. Further Analytical Equipment

CD and UV spectra were recorded with a J-815 spectropolarimeter (Jasco Germany, Groß-Umstadt, Germany) in methanol. Optical rotation was measured with a Jasco P-2000 polarimeter in chloroform.

#### 3.3.6. Analytical Data of *O*-tigloylcyclovirobuxeine-B (**1**)

**Figure A1:**
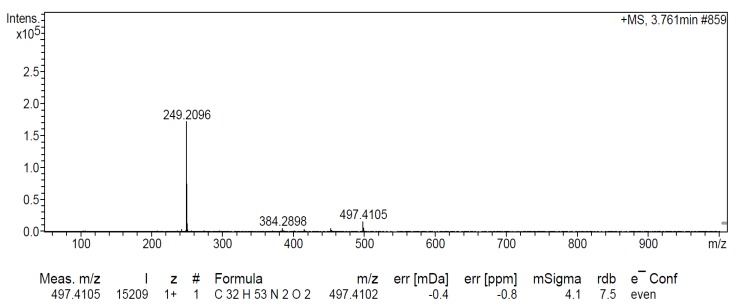
+ESI-QTOF mass spectrum of compound **1**.

**Figure A2:**
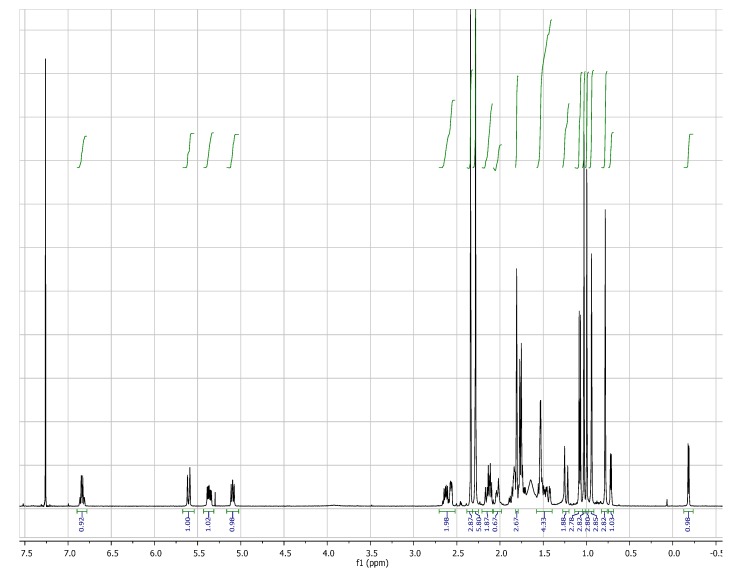
^1^H NMR spectrum (400 MHz, CDCl_3_) of compound **1**.

**Figure A3:**
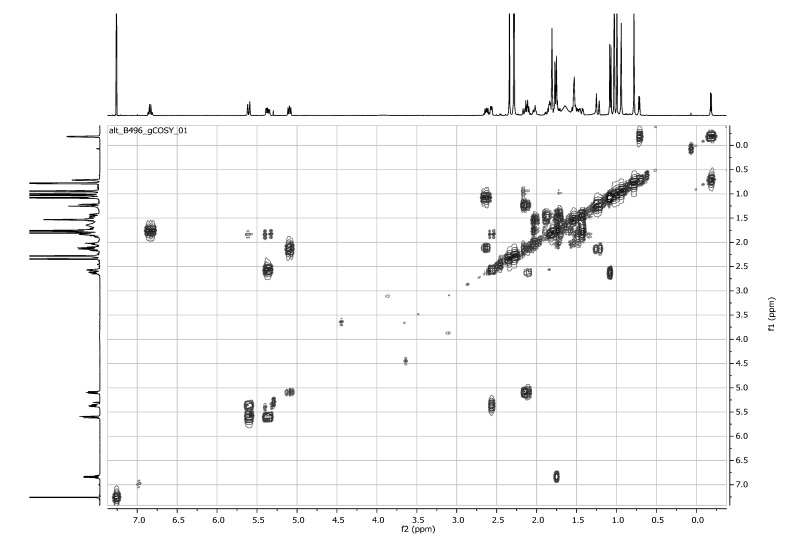
^1^H/^1^H COSY spectrum (400 MHz, CDCl_3_) of compound **1**.

**Figure A4:**
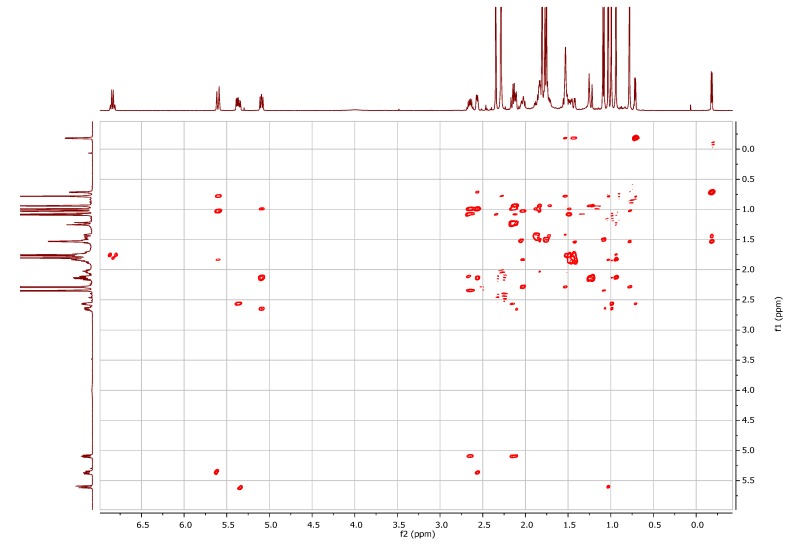
^1^H/^1^H NOESY spectrum (400 MHz, CDCl_3_) of compound **1**.

**Figure A5:**
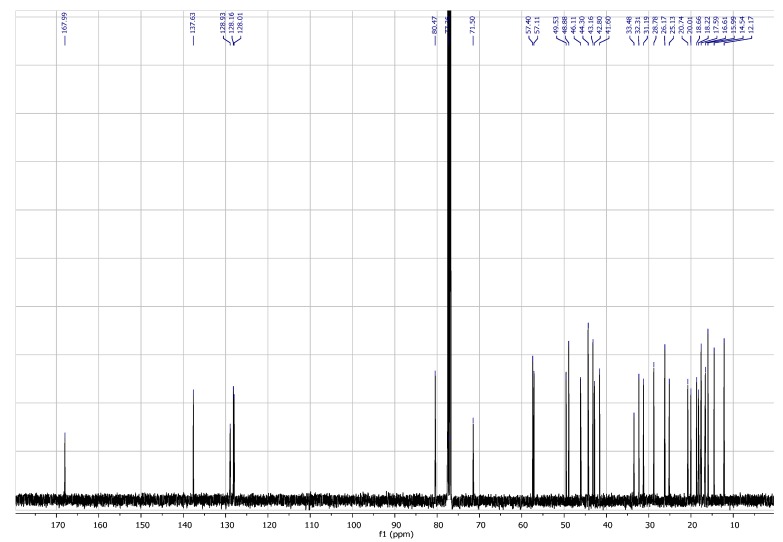
^13^C NMR spectrum (100 MHz, CDCl_3_) of compound **1**.

**Figure A6:**
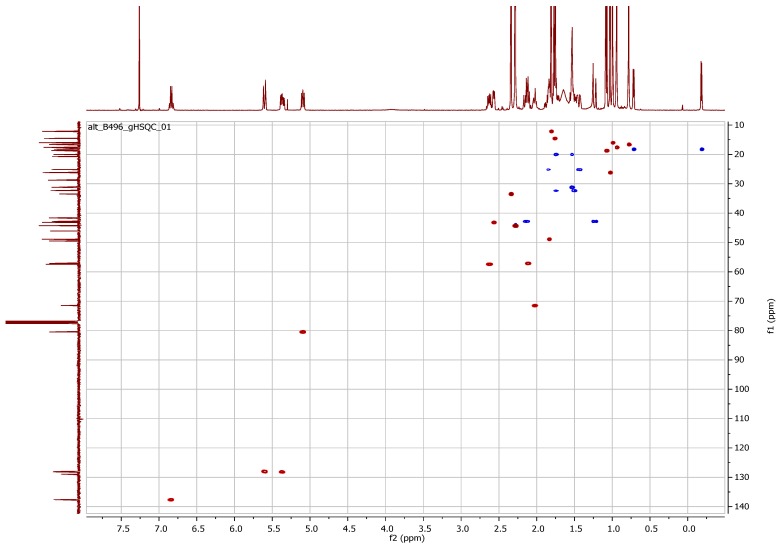
^1^H/^13^C HSQC spectrum (400/100 MHz, CDCl_3_) of compound **1**.

**Figure A7:**
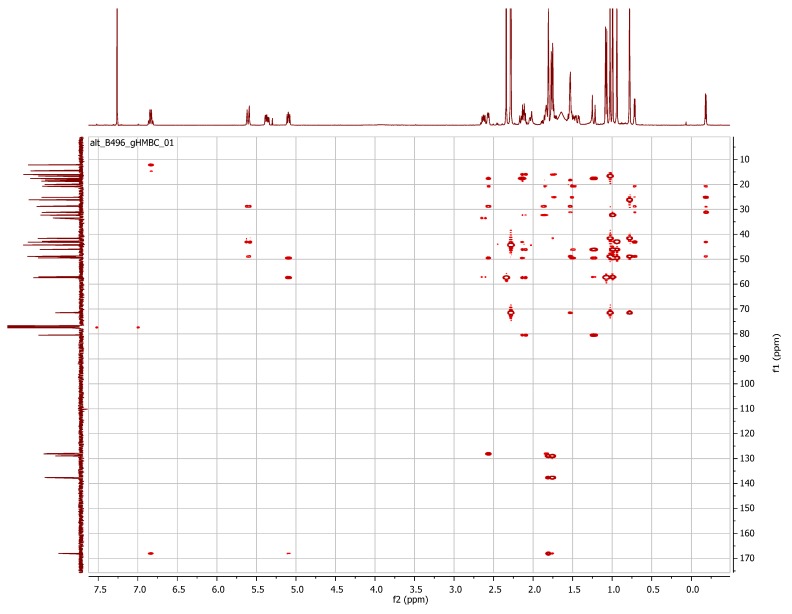
^1^H/^13^C HMBC spectrum (400/100 MHz, CDCl_3_) of compound **1**.

**Figure A8:**
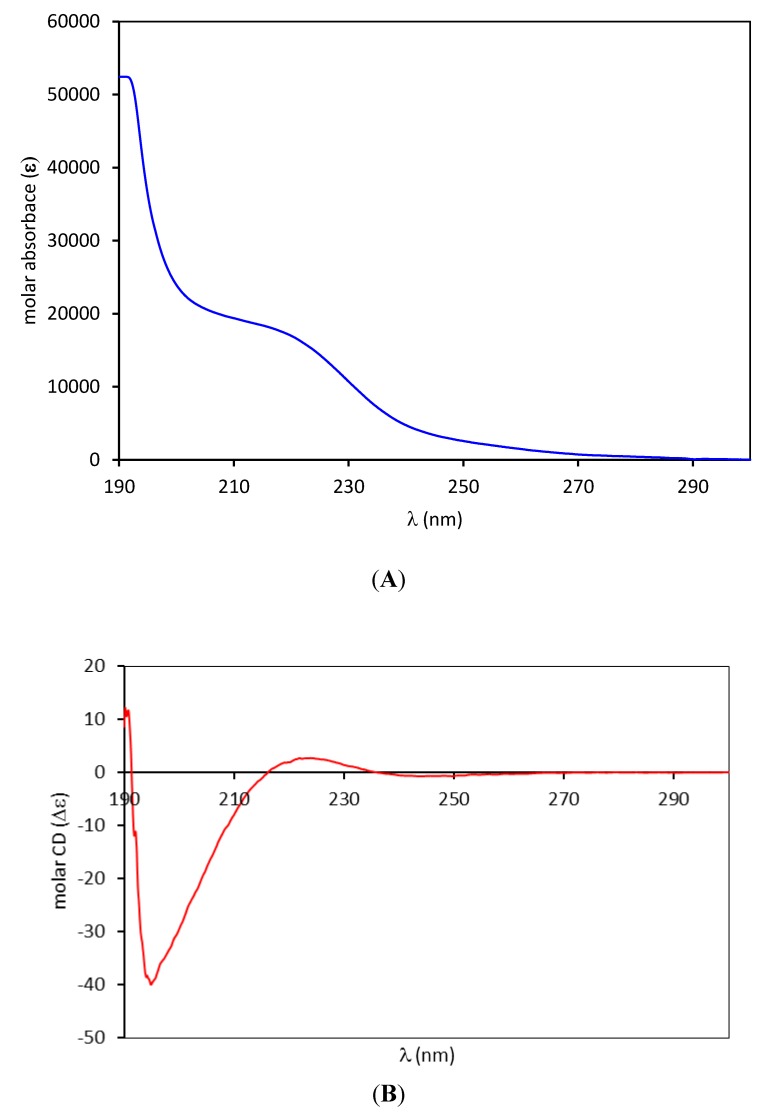
UV (**A**) and CD (**B**) spectra of compound **1** (MeOH).

[α]^20^_D_ −80° (*c* 0.27, CHCl_3_); UV (MeOH; λmax, log ε): 223 (sh, 4.2); 190 (4.8; last reading). CD (λ, Δε); 244 (−0.7); 223 (+2.7); 195 (−39.9); 190 (+11; last reading). +ESI-QTOF-MS (*m/z*) 497.4105 [M+H]^+^, 249.2096 [M+2H]^2+^ (calcd. for C_32_H_53_N_2_O_2_^+^: 497.4102^+^; for C_32_H_54_N_2_O_2_^2+^: 249.2088); ^1^H- and ^13^C-NMR data see Table 2. Mass-, 1D and 2D NMR as well as CD and UV spectra are shown in Figure A1, Figure A2, Figure A3, Figure A4, Figure A5, Figure A6, Figure A7 and Figure A8, appendix.

### 3.4. In Vitro Assays and IC_50_ Determination

The *in vitro* assays and the IC_50_ determination against *P. falciparum* (intraerythrocytic stages of strain NF54), *T. brucei rhodesiense* (STIB 900 strain, bloodstream trypomastigotes), *T. cruzi* (intracellular amastigotes, Tulahuen C4 strain), *L. donovani* (axenic amastigotes, strain MHOM-ET-67/L82) and L6 rat skeletal myoblast cells were performed as described previously [10].

## 4. Conclusions

*O*-tigloylcyclovirobuxeine-B (**1**) could be identified as the constituent mainly responsible for the antiplasmodial activity of *B. sempervirens* leaf extract. Due to the methodology applied here, targeting the most active fractions for antiplasmodial activity, it cannot be excluded that other related alkaloids also contribute to the overall effect of the total extract. The antiplasmodial potency of **1** after purification was slightly lower than that of the total alkaloid fraction. This may certainly be due to contributions from other compounds but could also simply be attributed to slight changes in the susceptibility of the parasites since several months passed between the first and last biological tests. It may thus be concluded that *O*-tigloylcyclovirobuxeine-B is the constituent of the investigated *B. sempervirens* leaves mainly responsible for the antiplasmodial activity and selectivity.

It is noteworthy that this antiplasmodial alkaloid also occurs at significant concentration in a decoction prepared according to the traditional mode of application of *B. sempervirens* leaves as antimalarial preparation. Our study thus provides support for the ethnomedicinal use of *B. sempervirens* in this indication.

Even though *B. sempervirens* has also been reported to possess some toxicity [5], the main antiplasmodial alkaloid **1** appears to be less toxic than others, e.g., those concentrated in fractions 98 and 116. The toxic and antiplasmodial activity of the extract can thus be separated from each other, which is an encouraging finding with respect to potential utility of **1** as a lead structure. Further studies on the *in vivo* potential and mechanism of action of *O*-tigloylcyclovirobuxeine-B are therefore warranted.

The antiplasmodial and antitrypanosomal activity as well as cytotoxicity were not correlated with each other during the bioactivity guided isolation. It is interesting to note that even though the crude extract did not show notable activity against *Tbr*, several of the spCCC subfractions displayed such activity at quite significant levels, e.g., subfraction E9 with an IC_50_ value 0.13 µg/mL against *Tbr* and an SI of 38. Isolation, identification and further evaluation of the antitrypanosomal potential of the constituents responsible for this activity are in progress.

Finally, it should not remain unmentioned, that in case *Buxus*-alkaloids turned out to be useful as starting material for new antimalarials, *B. sempervirens* would represent an extremely cost-efficient source of such compounds since it is very widespread in Europe as an ornamental plant. Immense quantities of leaves and young twigs would be available each year, simply from annual trimming of box hedges and trees.

## Appendixes

**Table A1:** *In vitro* growth inhibition [%] of *B. sempervirens* leaf extract and representative spiral-coil countercurrent chromatography (spCCC) subfractions of the alkaloid fraction. Each entry represents the mean of two independent measurements.

Tested sample	*P. falciparum*		*T. b. rhodesiense*		*T. cruzi*		*L. donovani*	
	10 µg/mL	2 µg/mL	10 µg/mL	2 µg/mL	10 µg/mL	2 µg/mL	10 µg/mL	2 µg/mL
spCCC subfractions elution phase								
Extract	99.1	20.8	16.8	0	17.6	0	43.0	0
62	100.0	66.7	100.0	96.5	49.7	5.3	70.8	25.0
82	100.0	77.2	100.0	84.8	82.5	10.0	13.6	24.2
98	100.0	99.7	100.0	100.0	87.5	16.8	16.1	21.9
116	99.7	93.3	100.0	100.0	102.1	52.1	12.1	14.7
130	100.0	60.4	100.0	100.0	95.3	43.7	10.4	15.7
142	99.5	60.6	100.0	100.0	85.8	13.9	11.8	12.5
146	99.8	51.8	100.0	100.0	85.8	26.0	0.0	3.6
spCCC subfractions extrusion phase								
E9	100.0	88.7	100.0	100.0	66.9	2.3	10.1	24.8
E53	100.0	99.8	100.0	92.8	26.6	3.3	17.8	26.6
E73	100.0	98.9	100.0	100.0	18.7	7.3	19.8	26.0
E97	98.9	27.4	100.0	88.8	12.6	0.0	23.9	27.6
E105	97.4	19.3	98.7	97.4	18.1	0.0	21.3	25.5
